# Associations among hair cortisol-to-DHEA ratio, psychological capital, harsh parenting, and internalizing problems in adolescence

**DOI:** 10.3389/fpsyg.2026.1899552

**Published:** 2026-09-15

**Authors:** Yapeng Liu, Xingliang Qi, Dan Tao, Yan Wu, Jin Yang, Huihua Deng

**Affiliations:** 1 College of Preschool Education, Nanjing Xiaozhuang University, Nanjing, China; 2 Institute of Child Development and Education, Southeast University, Nanjing, China; 3 School of Public Health, Southeast University, Nanjing, China; 4 Key Laboratory of Child Development and Learning Science (Southeast University), Ministry of Education, Nanjing, China

**Keywords:** adolescence, hair cortisol-to-DHEA ratio, harsh parenting, internalizing problems, psychological capital

## Abstract

**Introduction:**

Internalizing problems are increasingly prevalent among Chinese students in junior high school, and posing significant risks for long-term maladjustment. However, the mechanisms underlying these problems are unclear. The present study aimed to test the moderating effects of psychological capital and harsh parenting in the association between the hair cortisol-to-DHEA (C/D) ratio and internalizing problems in adolescence.

**Methods:**

282 junior high school students in China participated in this study (114 boys, 166 girls, and 2 with missing sex data; *M*age = 12.82 years). They provided hair samples and completed questionnaires on psychological capital, internalizing problems, harsh parenting of their parents, and control variables including sex, age, family socioeconomic status, negative life events, and body mass index. Hair concentrations of cortisol and DHEA were determined via liquid chromatography-tandem mass spectrometry.

**Results:**

The main effect of the hair C/D ratio on internalizing problems was not significant. The interaction between the C/D ratio and psychological capital was significant. Simple slope analysis revealed that a higher C/D ratio predicted fewer internalizing problems among adolescents with high psychological capital, but not among those with mean or low psychological capital. The three-way interaction involving harsh parenting was marginal after correction for multiple testing.

**Conclusion:**

Psychological capital moderates the association between the hair C/D ratio and adolescent internalizing problems. These findings highlight psychological capital as a potential intervention target for promoting adaptive stress responses in adolescents.

## 1 Introduction

Adolescence is marked by rapid changes in physical maturation, cognition, emotion, and social adaptation. Mental health issues have become a big problem for teenagers around the world. According to a recent survey by World Health Organization, 2025, about 15% of teenagers worldwide have serious mental health problems like depression and anxiety. This is especially severe in China, where an increasing number of junior high school students are struggling with feelings of depression, anxiety, and social withdrawal. These difficulties are collectively termed internalizing problems, referring to an inward-directed, overcontrolled pattern of emotional and behavioral problems (Madigan et al., 2013). Indeed, one study found that almost 22.7% of these students have symptoms that are severe enough to need professional help (Yu and Huang, 2023). These problems during adolescence do not go away when the teenagers grow up. They can lead to poor mental health, self-harm, and even suicidal thinking later in life (Geoffroy et al., 2025; Sivertsen et al., 2024). Thus, it is particularly important to understand the mechanisms behind adolescents’ internalizing problems. This understanding is helpful for developing targeted prevention and intervention strategies to reduce the risk of internalizing problems in adolescence.

The body operates a complex stress response system known as the hypothalamic-pituitary-adrenal (HPA) axis. Under stress, this system signals the adrenal cortex to release glucocorticoids, with cortisol serving as the primary one in humans. Cortisol can help the body adapt to challenge by adjusting its physiological state to meet situational demands, a process known as allostasis (McEwen, 1998; Smith and Vale, 2006). Disrupted HPA axis functioning has been linked to internalizing problems such as anxiety and depression (Carnegie et al., 2014; Li et al., 2022; Saridjan et al., 2014), but most of this work has relied on salivary cortisol. Salivary cortisol captures acute and diurnal fluctuations in HPA activity. However, it is sensitive to circadian rhythms and situational factors (Meyer and Novak, 2012).

Hair cortisol concentration (HCC) offers a complementary approach. Because hair grows at a fairly steady rate of about 1 cm per month, the proximal segment provides a retrospective record of cumulative cortisol exposure, with each centimeter capturing roughly 1 month of secretion (Wendsche et al., 2020). HCC is therefore one peripheral, retrospective marker that captures cumulative cortisol exposure over a defined period. Moreover, it is non-invasive and has high intra-individual reliability (Stalder and Kirschbaum, 2012). Although a growing number of studies have used hair cortisol to examine the link between HPA axis activity and internalizing problems, the findings have been mixed (Gerber et al., 2013; Lu et al., 2018; Sandstrom et al., 2020; Staufenbiel et al., 2013). Some studies have reported elevated HCC, whereas others have found reduced HCC or effects limited to certain subgroups or specific symptom dimensions. These mixed results may partly reflect the fact that different subtypes of internalizing behaviors are associated with distinct neuroendocrine profiles. Moreover, cortisol seldom acts in isolation, and examining it alongside other adrenal hormones may provide a fuller picture of how HPA axis activity relates to internalizing problems.

Dehydroepiandrosterone (DHEA) is co-secreted with cortisol by the adrenal gland (Hechter et al., 1997), but exerts opposing functional effects. Whereas cortisol suppresses inflammation and drives gluconeogenesis to sustain energy availability under stress, DHEA has anti-glucocorticoid properties (Kalimi et al., 1994; Kamin and Kertes, 2017). Because of this antagonistic relationship, the cortisol-to-DHEA ratio (C/D ratio) could offer a more reliable index of HPA axis functioning than either hormone considered alone. Thus, the C/D ratio has been proposed as an indicator of the net glucocorticoid profile under chronic stress (Ahmed et al., 2023; Kamin and Kertes, 2017). A high C/D ratio may reflect either elevated cortisol with normal DHEA, or normal cortisol with decreased DHEA (Ahmed et al., 2023). Both may indicate a neuroendocrine pattern relevant to social adjustment. Although the low C/D ratio has been more widely examined in relation to externalizing problems (Kamin and Kertes, 2017; Lee et al., 2024), evidence regarding internalizing problems is comparatively limited. Handley et al. (2023) found that elevated DHEA relative to average cortisol predicted heightened internalizing symptoms in maltreated children, but whether this pattern generalizes to other populations remains unclear.

The association between the C/D ratio and internalizing problems may depend on individual psychological resources that shape how adolescents respond to neuroendocrine stress. Psychological capital is a positive psychological state that develops over time and encompasses hope, optimism, self-efficacy, and resilience (Luthans et al., 2005). Conservation of resources theory proposes that individuals strive to acquire, maintain, and protect valued resources to cope with stress (Hobfoll and Shirom, 2001), and that those with more resources are less vulnerable to resource loss and tend to gain additional resources over time (Chen et al., 2015). As a key personal resource, psychological capital may buffer the effects of stress and reduce vulnerability to internalizing problems. Empirical evidence has also shown that high psychological capital was linked to reduced risk of internalizing problems (Song and Song, 2024; Zhongmin et al., 2022). Beyond this direct protective role, psychological capital may also moderate how neuroendocrine risk translates into internalizing symptoms. An unfavorable C/D ratio signals sustained HPA axis dysregulation, but this biological vulnerability need not inevitably lead to internalizing problems. Adolescents high in psychological capital tend to show stronger prefrontal regulation of limbic activity (Eaton et al., 2022; Müller-Pinzler et al., 2022; Yanagisawa et al., 2025), and the prefrontal cortex exerts top-down inhibitory control over HPA axis reactivity (Herman et al., 2005). The stress signal indexed by the C/D ratio may therefore be down-regulated before it gives rise to symptoms. Adolescents low in psychological capital, by contrast, lack this regulatory capacity, leaving the neuroendocrine signal more likely to manifest as emotional difficulties. However, no study has yet tested whether psychological capital moderates the link between the C/D ratio and internalizing problems.

Beyond psychological capital, how HPA-axis activity affects behavioral problems changes a great deal depending on the social context. For example, Cicchetti and Rogosch (2007) reported that higher morning cortisol predicted better resilient functioning among physically abused children, which is the opposite of the pattern in non-maltreated children. Similar context-dependent patterns appear with cumulative adversity. Iob et al. (2021) showed that cortisol was negatively related to depression among children exposed to three or more adverse childhood experiences, but this association was much weaker in children with fewer adversities. Tu et al. (2007) similarly found that in preterm infants subjected to elevated maternal stress and excessively intense interactive behaviors, increased basal cortisol concentrations were linked to diminished focused attention. Although these studies examined cortisol rather than the C/D ratio, they collectively suggest that the impact of HPA-axis activity on emotional and cognitive outcomes is strongly shaped by the caregiving context.

As a key feature of the caregiving environment, harsh parenting is broadly defined as parents’ hostile and forceful behavior toward children (Chang et al., 2003). Prior work has often associated harsh parenting with greater internalizing problems, though with some inconsistencies (Thompson et al., 2024; Zhang et al., 2024). Harsh parenting also appears to alter HPA axis functioning. Specifically, research has shown that maternal power control positively predicted cortisol reactivity in children (Azar et al., 2007). In contrast, overprotective parenting was linked to reduced C/D ratio (Ullmann et al., 2017). Moreover, when parental hostility is high, high cortisol reactivity is associated with greater internalizing symptom growth (Barrios et al., 2017). These findings suggest that the association between the C/D ratio and internalizing problems may be stronger under high harsh parenting.

According to conservation of resources theory, individuals with a richer resource pool are better able to protect existing resources and are less vulnerable to resource loss (Hobfoll, 2001; Chen et al., 2015). Adolescents with high psychological capital may therefore be less vulnerable to the resource-threatening effects of harsh parenting. Specifically, they may be better able to reinterpret threatening experiences, manage their distress, and mobilize social support through effective interpersonal engagement (Liu et al., 2024), weakening the link between harsh parenting and internalizing symptoms. In contrast, those with low psychological capital lack this buffer and are more vulnerable. Empirical evidence supports this view: high psychological capital has been found to reduce the adverse effects of parental psychological control and cumulative family risks on internalizing problems (Xiong et al., 2020; Zhou et al., 2025).

Building on the above reasoning, the moderating roles of psychological capital and harsh parenting may be interdependent, consistent with the biopsychosocial perspective that biological (C/D ratio), psychological (psychological capital), and social (harsh parenting) factors may interactively shape internalizing problems (Engel, 1977). Within the conservation of resources framework, harsh parenting can erode positive psychological resources over time (Ding et al., 2023), potentially triggering a resource loss spiral (Hobfoll, 2001) that weakens the buffering role psychological capital might otherwise provide. When harsh parenting is high, adolescents face a stressful caregiving environment that places greater demands on their coping capacities. For those who also have low psychological capital, internal resources are insufficient to offset environmental adversity and physiological stress, potentially amplifying the risk associated with an unfavorable C/D ratio. When harsh parenting is low, the environment itself poses minimal threat and may be unlikely to trigger the translation of an unfavorable C/D ratio into internalizing problems.

In addition, most prior research focused on Western samples. Chinese adolescents grow up in a cultural context marked by collectivist values and distinctive parenting norms that emphasize training and parental involvement (Chao, 1994). Harsh parenting is perceived as more normative in China (Zhang et al., 2024). These factors may influence both HPA axis activity and the psychological meaning of harsh parenting. Examining these associations in a Chinese sample provides an opportunity to test whether findings from Western contexts generalize cross-culturally.

Based on the above, the present study tested the following hypotheses in a Chinese sample. Hypothesis 1: The hair C/D ratio is negatively associated with internalizing problems in adolescents. We note that direct evidence for this prediction is limited and largely drawn from maltreated samples (Handley et al., 2023), so this hypothesis should be regarded as exploratory. Hypothesis 2: Psychological capital moderates the association between the hair C/D ratio and internalizing problems, such that this negative association is stronger among adolescents with higher psychological capital. Hypothesis 3: Harsh parenting moderates the association between the hair C/D ratio and internalizing problems, such that this negative association is weaker among adolescents exposed to higher levels of harsh parenting. In addition, we conducted an exploratory analysis to examine whether psychological capital and harsh parenting jointly moderated the link between the hair C/D ratio and internalizing problems (i.e., a three-way interaction). Given the absence of prior empirical work on this specific combination and the well-documented power limitations of three-way interaction tests, this analysis was treated as hypothesis-generating rather than hypothesis-confirming.

## 2 Materials and methods

### 2.1 Participants

A total of 320 seventh-grade students were recruited from a junior high school in Jiangsu Province, China. Participants needed to meet the following eligibility criteria: (1) no diagnosed chronic physical or mental health conditions; (2) in the past 6 months, without any use of hormonal medications, including oral contraceptive; and (3) no chemical hair treatments (e.g., dyeing or perming) during the same period. The Institutional Review Board of Southeast University Zhongda Hospital granted ethical approval for the present study (No. 2014ZDSYLL004.0; January 29, 2014). All procedures performed in this study were in accordance with the principles of the Declaration of Helsinki.

Of the 320 students, 14 were excluded because their HCC or DHEA concentrations exceeded ± 3 *SD* from the mean. Another 24 students were also excluded for incomplete data on internalizing problems, harsh parenting, or psychological capital. The final sample comprised 282 participants (*Mage* = 12.82, *SD* = 0.631, *range* = 12–15 years). Of these, 114 were male (40.4%), 166 were female (58.9%), and 2 had missing sex data (0.7%). In terms of parental education, 38.3% fathers and 35.8% mothers had a college or associate degree. Approximately 47.9% families reported an annual household income ranging from ¥100,000 to ¥200,000. The summary statistics for the sample were presented in Table 1.

**TABLE 1:** Sample characteristics and descriptive statistics (*n* = 282).

Variable	*N*	Percentage (%)	*M*	*SD*	*Range*	*Median (IQR)*
Sex						
Boys	114	40.4%	–	–	–	–
Girls	166	58.9%	–	–	–	–
Unreported	2	0.7%			–	–
Parental education						
Primary school or below	7 (5)	2.5% (1.8%)	–	–	–	–
Junior high school	51 (45)	18.1% (16.0%)	–	–	–	–
High school	71 (82)	25.2% (29.1%)	–	–	–	–
Bachelor’s or associate degree	108 (101)	38.3% (35.8%)	–	–	–	–
Master’s degree or higher	38 (43)	13.5% (15.2%)	–	–	–	–
Unreported	7 (6)	2.5% (2.1%)	–	–	–	–
Annual household income						
≤ ¥100,000	61	21.6%	–	–	–	–
¥100,000 to ¥200,000	135	47.9%	–	–	–	–
¥200,000 to ¥300,000	53	18.8%	–	–	–	–
≥ ¥300,000	25	8.9%	–	–	–	–
Unreported	8	2.8%	–	–	–	–
SES	–	–	−0.070	2.250	−6.20 to 5.14	−0.100 (2.83)
Age	–	–	12.820	0.631	12–15	13.000 (1.00)
BMI	–	–	18.529	3.142	13.33–28.23	17.960 (3.83)
Stressful life events	–	–	1.821	0.615	1.00–4.00	1.730 (0.88)
Internalizing problems	–	–	0.252	0.276	0.00–1.26	0.160 (0.32)
HCC (pg/mg)	–	–	7.366	5.521	1.011–30.541	5.656 (6.407)
DHEA (pg/mg)	–	–	3.827	1.853	1.152–14.163	3.411 (2.251)
Ln C/D ratio	–	–	0.517	0.838	−1.43 to 2.57	0.520 (1.20)
Psychological capital	–	–	5.363	0.925	2.69–7.00	5.480 (1.40)
Harsh parenting	–	–	1.483	0.461	0.97–3.06	1.330 (0.54)

For parental education, values outside parentheses were for mothers, values inside parentheses were for fathers; Sex: boy was coded as 0 and girl was coded as 1; BMI, body mass index; Age, adolescent age; SES, social economic status; HCC, hair cortisol concentration; DHEA, Dehydroepiandrosterone; Ln C/D ratio, log-transformed the cortisol-to-DHEA ratio.

### 2.2 Procedure

Following informed consent, participants completed a battery of self-report questionnaires assessing internalizing problems, harsh parenting, psychological capital, negative life events, and control variables including age, sex, body mass index (BMI), annual household income, and parents’ educational levels. Hair samples were cut from the posterior vertex of the head by trained barbers at the participants’ school. From each participant, two small tufts of hair (about 25 mm in length) were clipped as close to the scalp as possible. The proximal 1-cm length (at least 50 mg) was then saved for analysis. Hair samples were wrapped in aluminum foil, placed in paper envelopes, and stored in a dark, ventilated area at room temperature. All samples were assayed within 5 months of collection, well within the 6-month window recommended to minimize analyte degradation (Huthsteiner et al., 2025).

### 2.3 Measures

#### 2.3.1 Measures of internalizing problems

The Chinese version of the Youth Self-Report was used to measure internalizing problems (Wang et al., 2013). Its reliability and validity have been established in Chinese adolescent samples. It contains 119 items across eight subscales. Items were rated on a 3-point scale (0 = not true, 1 = somewhat or sometimes true, 2 = very true or often true). Internalizing problems are evaluated using the Anxious/Depressed (e.g., “I feel worthless or inferior”), Withdrawn/Depressed (e.g., “avoiding eye contact”), and Somatic Complaints (e.g., “frequent headaches”) subscales. The remaining five subscales assess externalizing problems and problems that are neither internalizing nor externalizing, which were not the focus of the present study. Higher composite scores reflect greater symptom severity. The Cronbach’s α coefficient for the composite internalizing problems score was 0.95.

#### 2.3.2 Hair C/D ratio

The proximal 1-cm hair segment was taken for steroid analysis. This segment represents approximately 1 month of cumulative steroid secretion. The assay procedure has been detailed in a previous report (Chen et al., 2014). In brief, samples were cleaned by washing with methanol, air-dried, and minced into small segments. The approximately 20 mg aliquots were weighed into centrifuge tubes, followed by the addition of 1.8 mL of methanol and 20 μL of internal standards. The mixture was incubated at room temperature for 24 h. After centrifugation, 1.6 mL of supernatant was collected into a new centrifuge tube and dried at 50 °C under a gentle nitrogen stream (purity ≥ 99.9%). The residue was reconstituted in water/methanol mixture and purified using a Pro Elut C18 solid-phase extraction column (Dikma Technologies). Hair cortisol and DHEA concentrations were quantified by liquid chromatography–tandem mass spectrometry (LC-MS/MS) as described by Mwanza’s group (Mwanza et al., 2016). Both intra- and inter-assay coefficients of variation (CVs) were below 10%, and analyte recovery ranged from 92.5 to 114.3%.

#### 2.3.3 Harsh parenting

Participants completed the Chinese version of the Egna Minnen av Barndoms Uppfostran (EMBU; Yue et al., 1993). The paternal form comprises 58 items across six dimensions. The maternal form has 57 items across five dimensions with Excessive Interference and Overprotection merged into a single subscale. Each item was rated by students on a 4-point scale from 1 (never) to 4 (always). Given the strong correlations between the Punishment/Harshness (e.g., “My parents often beat me.”) and Rejection/Denial (e.g., “My parents often told me that they did not like my behavior at home.”) subscales (rs = 0.80 and 0.84 for the maternal and paternal scales, respectively), these two subscales were combined into a single harsh parenting composite, following the approach used by Kennedy et al. (2004). The composite scale had high internal consistency (Cronbach’s α = 0.94 for both fathers and mothers). Maternal score was strongly correlated with paternal score (*r* = 0.83), so they were averaged to form a single parental index.

#### 2.3.4 Psychological capital

To assess psychological capital, the Positive Psychological Capital Questionnaire was used (Zhang et al., 2010). This 26-item scale covers hope (e.g., “I am energetically pursuing my goals.”), self-efficacy (e.g., “I am more insightful and capable than most people.”), resilience (e.g., “I seldom care about life’s frustrations.”), and optimism (e.g., “I usually expect the best.”) subscales. Each item was rated by participants on a 7-point scale (1 = strongly disagree; 7 = strongly agree). The Cronbach’s α coefficient for the present sample was 0.94.

### 2.4 Control variables

*Family socioeconomic status (SES)*. SES was measured through two primary indicators: the educational levels achieved by parents and the yearly household income. Each indicator was standardized within sample into a Z-score. Subsequently, following Liang et al. (2021), a composite SES index was computed by summing these equally weighted Z-scores: Z SES = Z father’s education + Z mother’s education + Z household income.

*Negative Life Events.* The Adolescent Self-Rating Life Events Checklist was used to evaluate negative events that occurred in the past year (Xin and Yao, 2015). This checklist includes 27 items. Participants assessed each item on a scale from 1 (no impact) to 5 (extreme impact). In this study, the Cronbach’s α was 0.95.

*Demographic Variables.* Students’ age was noted in years. Sex was coded with 0 representing boys and 1 representing girls. We calculated BMI as weight (kg) divided by height squared (m^2^).

### 2.5 Statistical analysis

Preliminary data management was conducted in SPSS 27.0 for Windows. The proportion of missing data ranged from 0 to 5.8% across study variables. Little’s MCAR test did not show significance (*p* > 0.05), supporting the missing-completely-at-random assumption. Consequently, we addressed the missing values through multiple imputations, conducting five imputations.

The hair C/D ratio was computed by dividing HCC concentration by their DHEA concentration for each participant, with both measurements in pg/mg. Kolmogorov–Smirnov tests revealed significant deviations from normality in the C/D ratio (*p* < 0.001; skewness = 2.26, kurtosis = 6.43). Thus, it was log-transformed (Cicchetti and Rogosch, 2007; Lee et al., 2024). Following this transformation, the distribution no longer significantly departed from normality (*p* > 0.05; skewness = 0.08, kurtosis = −0.40). Outliers on continuous variables (beyond ± 3 *SD*) were removed (Ji et al., 2021).

Initially, descriptive statistics were performed. Categorical variables were summarized using frequencies and percentages, while continuous variables were characterized by means, standard deviations, range, and median (IQR). To investigate sex differences in adolescents’ psychological capital, internalizing issues, and hair C/D ratio, as well as the variation in parental harsh parenting based on child sex, independent-samples *t*-tests were utilized. Following this, Pearson correlations were calculated for all study variables, excluding sex. To account for multiple comparisons, all correlation *p*-values were adjusted using the Benjamini-Hochberg false discovery rate (FDR) procedure (Benjamini and Hochberg, 1995).

In R, regression analyses were conducted using ordinary least squares (OLS) estimation with the *lm()* function. Heteroscedasticity-consistent standard errors (HC3) were also computed using the sandwich package (Zeileis, 2004) as a robustness check. To control for multiple testing, *p*-values were adjusted using the FDR correction. Prior to the regression analysis, all continuous predictors were mean-centered. In this model, control variables including sex, age, BMI, negative life events, and SES were entered first. Next, the main effects of Ln C/D ratio, psychological capital, and harsh parenting were entered into the next step. Three two-way interactions (Ln C/D ratio with psychological capital, Ln C/D ratio with harsh parenting, and psychological capital with harsh parenting) were added in the next step. Finally, the three-way interaction among Ln C/D ratio, psychological capital, and harsh parenting was entered. Multicollinearity was evaluated via variance inflation factors (VIFs), with all VIFs < 1.50.

## 3 Results

### 3.1 Common method bias

We employed the Harman’s single-factor test to check common method bias. Without rotation, we found 24 factors with eigenvalues above 1. The first factor accounted for 19.55% of the total variance, significantly less than the 50% threshold. It implied that there was no major bias in common method in this study.

### 3.2 Descriptive statistics

Table 1 presented the means and standard deviations of internalizing problems, harsh parenting, psychological capital, Ln C/D ratio, stressful life events, and SES. Significant sex differences emerged from independent-samples *t*-tests. Compared to boys, girls had significantly higher levels of internalizing problems [*t*_(274)_ = −2.498, *p* = 0.013]. Boys experienced harsher parenting than girls [*t*_(270.826)_ = 3.165, *p* = 0.002]. These results are shown in Table 2.

**TABLE 2:** Sex differences in key study variables.

Variables	Boys (*M ± SD*)	Girls (*M ± SD*)	*t*	*df*	*p-*value
Internalizing problems	0.200 ± 0.250	0.284 ± 0.286	−2.498	274	0.013
Ln C/D ratio	0.567 ± 0.899	0.489 ± 0.797	0.767	278	0.444
Psychological capital	5.292 ± 0.990	5.439 ± 0.847	−1.329	277	0.185
Harsh parenting	1.587 ± 0.501	1.405 ± 0.415	3.165	270.826	0.002

### 3.3 Correlation analysis

Results of Pearson correlations, corrected for multiple comparisons using the Benjamini-Hochberg FDR procedure, were presented in Table 3. The results indicated that internalizing problems exhibited a negative link with psychological capital and a positive link with harsh parenting. Psychological capital was negatively related to harsh parenting. Among the control variables, BMI exhibited a negative correlation with psychological capital and a positive correlation with harsh parenting. Negative life events were correlated with higher internalizing problems and harsh parenting, but lower psychological capital. Socioeconomic status was positively linked with psychological capital.

**TABLE 3:** Correlation coefficients of variables.

Variables		1	2	3	4	5	6	7	8
1. age (years)	*r*	–	–	–	–	–	–	–	–
	*p*	–							
2. BMI	*r*	0.088							
	*p*	0.250	–						
3. Stressful life events	*r*	0.007	0.063						
	*p*	0.906	0.450	–					
4. SES	*r*	0.057	−0.016	−0.128					
	*p*	0.480	0.821	0.075	–				
5. Internalizing problems	*r*	−0.062	0.018	0.543	−0.124				
	*p*	0.450	0.821	< 0.001	0.083	–			
6. Ln C/D ratio	*r*	−0.065	0.068	0.017	−0.110	0.048			
	*p*	0.450	0.250	0.821	0.134	0.572	–		
7. Psychological capital	*r*	−0.043	−0.179	−0.409	0.167	−0.379	−0.130		
	*p*	0.602	0.009	< 0.001	0.016	< 0.001	0.075	–	
8. Harsh parenting	*r*	0.133	0.192	0.365	0.028	0.253	0.035	−0.376	
	*p*	0.705	0.005	< 0.001	0.752	< 0.001	0.686	< 0.001	–

Ln C/D ratio, log-transformed the cortisol-to-DHEA ratio; Reported *p*-values were adjusted for multiple comparisons using the Benjamini-Hochberg false discovery rate (FDR) procedure.

### 3.4 Regression analysis

Hierarchical multiple regression was conducted to test the interaction of hair C/D ratio with psychological capital and harsh parenting in predicting internalizing problems. Results remained unchanged when heteroscedasticity-consistent standard errors (HC3) were applied (see Supplementary Material). As shown in Table 4, psychological capital negatively predicted internalizing problems (β = −0.136, *p* = 0.021), however, this effect became marginal after FDR correction (*p*_FDR_ = 0.068). Ln C/D ratio and harsh parenting were not significantly associated with internalizing problems (*ps* = 0.454 and 0.302).

**TABLE 4:** Hierarchical regression coefficients predicting internalizing problems.

Predictors	*Δ R^2^*	*B*	*SE*	β	*p-*value	*p*_*FDR*_	*95%CI*
Step 1	0.320**						
Sex		0.102	0.029	0.189	0.001	0.002	[0.045, 0.160]
Age		−0.012	0.022	−0.027	0.594	0.702	[−0.054, 0.031]
SLE		0.210	0.025	0.479	< 0.001	<0.001	[0.161, 0.258]
SES		−0.004	0.006	−0.035	0.500	0.654	[−0.016, 0.008]
BMI		0.000	0.004	0.004	0.981	0.982	[−0.008, 0.008]
Step 2	0.026*						
C/D		−0.013	0.018	−0.042	0.454	0.654	[−0.048, 0.022]
PsyCap		−0.040	0.017	−0.136	0.021	0.068	[−0.074, −0.006]
HP		0.035	0.034	0.060	0.302	0.561	[−0.032, 0.103]
Step 3	0.027*						
C/D × PsyCap		−0.064	0.018	−0.198	< 0.001	0.002	[−0.099, −0.028]
C/D × HP		−0.050	0.047	−0.059	0.285	0.561	[−0.142, 0.042]
PsyCap × HP		0.022	0.032	0.035	0.503	0.654	[−0.042, 0.085]
Step 4	0.012*						
C/D × PsyCap × HP		−0.116	0.052	−0.127	0.028	0.072	[−0.219, –0.013]

Covariates: Sex, Age, SLE, SES, and BMI; SLE, stressful life events; C/D, Ln C/D ratio; PsyCap, psychological capital; HP, harsh parenting; *p < 0.05; ** *p* < 0.01.

Further, the interaction between Ln C/D ratio and psychological capital was significant (β = −0.198, *p* < 0.001), and this effect remained significant after FDR correction (*p*_FDR_ = 0.002). This significant interaction was probed using simple slope analysis, with conditional effects estimated at -1 *SD*, the mean, and +1 *SD* of psychological capital. After FDR correction, the Ln C/D ratio significantly and negatively predicted internalizing problems among adolescents with high psychological capital (*slope* = −0.071, *t* = −3.015, *p*_FDR_ = 0.008), but not at the mean (*slope* = −0.013, *t* = −0.727, *p*_FDR_ = 0.468) and low (*slope* = 0.045, *t* = 1.822, *p*_FDR_ = 0.105) levels. These results are presented in Figure 1.

**FIGURE 1:**
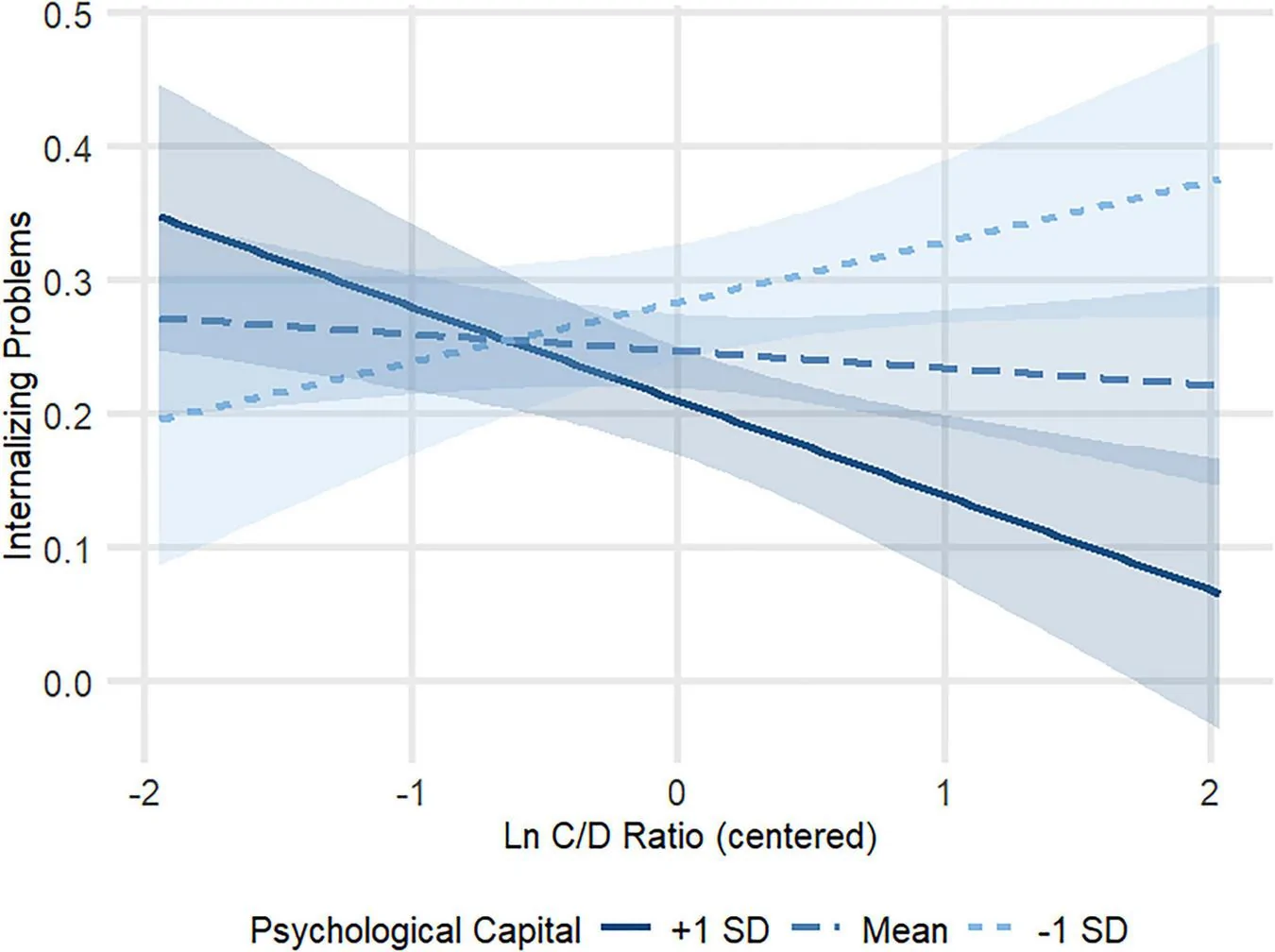
Interaction among the Ln C/D ratio and psychological capital in predicting adolescent internalizing problems. PsyCap refers to psychological capital. Shaded areas represent 95% confidence intervals.

Finally, the three-way interaction among Ln C/D ratio, psychological capital, and harsh parenting was significant (β = −0.127, *p* = 0.028). However, this effect became marginal after FDR correction (*p*_FDR_ = 0.072). A sensitivity power analysis was conducted for the three-way interaction term using G*Power 3.1 (linear multiple regression, *R*^2^ increase). With the current sample size (*N* = 282), α = 0.05, *power* = 0.80, and 12 predictors in the full model, the minimum detectable effect size was *f^2^* = 0.029 (corresponding to *ΔR^2^* ≈ 0.028). The observed effect size of the three-way interaction (*f^2^* = 0.019, *ΔR^2^* = 0.012) fell below this threshold, indicating that the present study was underpowered to reliably detect the three-way interaction. This finding should therefore be interpreted with caution. As an exploratory step, we conducted simple slope analyses to further probe this interaction. Detailed results were presented in the Supplementary Material.

## 4 Discussion

The present study examined how the hair C/D ratio, psychological capital, and harsh parenting jointly relate to internalizing problems among Chinese adolescents. Overall, the C/D ratio interacted with psychological capital to predict internalizing problems, and this effect remained robust after correction for multiple comparisons. Among adolescents with high psychological capital, a higher C/D ratio was associated with fewer internalizing problems. The three-way interaction involving harsh parenting, by contrast, became only marginal after correction.

In the present study, the main effect of the hair C/D ratio on internalizing problems was not significant. This finding is inconsistent with Chen et al. (2015), who reported a positive association between the salivary cortisol/DHEA-s ratio and internalizing symptoms. This discrepancy may be partly attributable to differences in the temporal window captured by each method. Salivary cortisol reflects momentary HPA axis activity and is sensitive to circadian rhythms and situational factors at the time of sampling (Meyer and Novak, 2012), whereas hair cortisol concentration measured from the proximal 1-cm segment indexes cumulative HPA axis activity over approximately the preceding month (Wendsche et al., 2020). These two approaches therefore capture different aspects of HPA axis functioning, which may account for the divergent findings. Turning to the present study specifically, the non-significant main effect observed here does not necessarily indicate the absence of a true association. The relationship between the hair C/D ratio and internalizing problems may be conditional on individual factors, as the significant interaction effects in this study suggest.

The interaction between the hair C/D ratio and psychological capital was significant after FDR correction. The results indicated that a higher C/D ratio was associated with fewer internalizing problems specifically among adolescents with high psychological capital, whereas no reliable association emerged at mean or low levels of psychological capital. This pattern is broadly consistent with prior evidence suggesting that an elevated C/D ratio may reflect adaptive rather than maladaptive neuroendocrine functioning under certain conditions. Cicchetti and Rogosch (2007) reported that morning and afternoon salivary C/D ratios were positively related to resilient functioning. Handley et al. (2023) found that maltreated children with an increased cortisol-to-DHEA profile were rated as more likeable by peers, whereas those with a comparatively lower ratio (average cortisol, high DHEA) showed more internalizing symptoms. Both of these prior studies indexed acute HPA activity via salivary cortisol, whereas the present study indexed cumulative secretion via hair cortisol. The convergence of this general pattern across methodologically distinct approaches lends some credibility to the possibility that an elevated C/D ratio may reflect adaptive neuroendocrine mobilization under certain conditions.

One possible explanation for this result in present study may involve prefrontal regulatory capacity and cognitive strategies. Adolescents with high psychological capital have more personal resources and are better able to generate additional resources (Chen et al., 2015), thereby possessing greater self-efficacy, optimism, hope, and resilience. Resilience, self-efficacy, and optimism have each been associated with greater prefrontal-limbic connectivity (Eaton et al., 2022; Müller-Pinzler et al., 2022; Yanagisawa et al., 2025). The prefrontal cortex is known to exert inhibitory control over the amygdala and HPA axis activity (Herman et al., 2005). Moreover, individuals with stronger psychological capital tend to manage stress more effectively through cognitive strategies such as reappraisal (Liu et al., 2024). Acute stress research has shown that reappraising physiological arousal as a coping resource instead of a threat signal can redirect stress-related mobilization toward focused attention and active problem-solving (Jamieson et al., 2012, 2013). Notably, Akinola et al. (2016) found that cortisol increases were positively related to performance when participants appraised their anxiety as beneficial. We tentatively propose that a similar process may operate over longer timescales. Specifically, adolescents with higher psychological capital may habitually reappraise stress arousal as facilitative during everyday stressful encounters. Through repeated use, this tendency could be gradually reflected in how they respond to the chronic activation captured by an elevated hair C/D ratio, such that the mobilization aligns with goal-oriented effort rather than emotional disturbance. In this way, the physiological mobilization indexed by a higher C/D ratio may serve an adaptive function for these adolescents, and this may partly account for the fewer internalizing problems observed in this group. Adolescents with mean or low psychological capital may lack the reappraisal capacity and prefrontal regulatory resources needed to link cortisol-driven arousal with organized coping. This may explain why the C/D ratio was not negatively associated with internalizing problems in these groups. Given that the reappraisal mechanism has only been documented in acute laboratory settings and that the present study employed a cross-sectional design, this interpretation remains speculative. More longitudinal studies tracking both appraisal orientations and neuroendocrine indicators over time are needed to test it directly.

In addition, Handley et al. (2023) found that maltreated children with an increased cortisol-to-DHEA profile were rated as more likeable by peers. Yet the present study did not find a significant interaction between the hair C/D ratio and harsh parenting. One possible explanation draws on the notion of “hidden talents,” which holds that children who grow up in stressful and unpredictable environments may develop specialized skills that carry adaptive value in those settings (Ellis et al., 2022). Seen this way, the elevated cortisol-to-DHEA profile in maltreated children is not necessarily a maladaptive state (Handley et al., 2023). It may instead co-occur with stronger social skills, which could explain why these children were rated as more likeable by peers. Maltreatment typically involves severe interpersonal threat, whereas harsh parenting rarely reaches this level. According to parental acceptance-rejection theory, what shapes a child’s adjustment is not the parenting behavior itself, but whether the child construes it as rejection or as something more normative and even caring (Rohner, 1986; Rohner et al., 2005). Where harsh parenting is culturally commonplace, adolescents may interpret it as reasonable rather than threatening, and this appraisal may buffer its psychological impact. Such a dynamic is especially relevant in Chinese cultural contexts, where harsh parenting has long been normalized and often understood as an expression of care (Zhang et al., 2024), as reflected in the folk saying “beating is closeness, scolding is love” (Liu and Wang, 2018). Consistent with this, Liu and Wang (2018) reported a substantially weaker link between harsh discipline and behavioral problems among adolescents who appraised it as normative. These considerations may together help account for the absence of a moderating effect of harsh parenting on the association between the C/D ratio and internalizing problems in the present sample.

Previous research has linked the C/D ratio to both externalizing and internalizing problems (Kamin and Kertes, 2017; Lee et al., 2024), but little is known about the conditions under which this association holds. The present study contributes to this area by demonstrating that the association between the hair C/D ratio and internalizing problems was significant only among adolescents with high psychological capital. However, several limitations warrant consideration:

A primary limitation concerns the confounding role of pubertal development. DHEA rises sharply and variably during adrenarche. An adolescent at a more advanced adrenarcheal stage would have higher DHEA and a lower C/D ratio regardless of chronic stress, potentially either generating or masking the C/D ratio effects observed here. We controlled for age, sex, and BMI in an attempt to account for pubertal variation. However, these are imperfect proxies that cannot fully capture individual differences in pubertal timing. Similar reliance on such proxies has been reported in other studies examining cortisol and DHEA in comparable age groups (e.g., Goodyer et al., 2000; Handley et al., 2023), though we recognize this does not diminish the importance of the issue. Future studies should incorporate direct indices such as Tanner staging or menarcheal status to disentangle adrenarcheal maturation from stress-related variation in the C/D ratio.

Second, the present study used a cross-sectional design to examine the interacting effect of hair C/D ratio with psychological capital and harsh parenting in adolescents’ internalizing problems. We therefore cannot make causal claims, and longitudinal work is needed to establish the temporal ordering of these associations. A related measurement constraint is that we assayed only the proximal 1-cm hair segment, which reflects cumulative cortisol and DHEA secretion over roughly the past month. This segment cannot speak to longer-term exposure or to how hormone secretion changes over time, both of which would require analyzing multiple segments along the hair shaft. In addition, hair steroid concentrations may be affected by several unmeasured factors, such as hair-washing frequency, UV exposure, hair pigmentation, and individual differences in hair growth rate. Thus, the hair-derived C/D ratio values should be interpreted with caution.

Third, the three-way interaction did not remain significant after correction for multiple comparisons, and a sensitivity analysis indicated that the study was underpowered to reliably detect an effect of this size. This finding should therefore be regarded as preliminary and requires replication in larger samples.

Finally, the study was conducted in China, Jiangsu Province, where the per capita disposable income has consistently exceeded the national average over the past years (National Bureau of Statistics of China, 2024). Moreover, all participants were recruited from a single school within this region, further constraining the representativeness of the sample. Caution is therefore warranted when generalizing these findings. Future studies should investigate the mechanisms underlying adolescent internalizing problems in more diverse samples across multiple schools and regions.

## 5 Conclusion

This study examined the interplay between the hair C/D ratio, psychological capital, and harsh parenting in relation to internalizing problems among adolescents. The key finding is that the association between the hair C/D ratio and internalizing problems was conditional on the level of psychological capital. Specifically, a higher C/D ratio was associated with fewer internalizing problems only among adolescents with high psychological capital. For those with mean or low psychological capital, no such association emerged. This pattern is consistent with the possibility that psychological capital may be associated with adolescents’ capacity to channel stress-related arousal toward adaptive coping. These findings highlight the importance of considering psychological capital as a moderator of the stress–psychopathology link and point to the potential value of fostering psychological resources in adolescent mental health interventions.

## Data Availability

The original contributions presented in this study are included in this article/Supplementary material, further inquiries can be directed to the corresponding authors.
