# Supplementary material for: Associations among hair cortisol-to-DHEA ratio, psychological capital, harsh parenting, and internalizing problems in adolescence
Source: Front Psychol. 2026 Sep 15;17:1899552. doi: 10.3389/fpsyg.2026.1899552 (PMC13619371; doi:10.3389/fpsyg.2026.1899552)
Supplement: Supplementary file 1 [file Table_1.docx]

**TABLE 5** Hierarchical regression coefficients predicting internalizing problems with OLS and heteroscedasticity-consistent (HC3) standard errors.

| **Predictors** | ***B*** | ***SE_OLS*** | ***p _OLS*** | ***p*_FDR** | ***SE_HC3*** | ***p_HC3*** |
| --- | --- | --- | --- | --- | --- | --- |
| Step 1 |  |  |  |  |  |  |
| Sex | 0.102 | 0.029 | 0.001 | 0.002 | 0.028 | <0.001 |
| Age | -0.012 | 0.022 | 0.594 | 0.702 | 0.025 | 0.644 |
| SLE | 0.210 | 0.025 | <0.001 | <0.001 | 0.029 | <0.001 |
| SES | -0.004 | 0.006 | 0.500 | 0.654 | 0.006 | 0.516 |
| BMI | <0.001 | 0.004 | 0.981 | 0.982 | 0.003 | 0.979 |
| Step 2 |  |  |  |  |  |  |
| C/D | -0.013 | 0.018 | 0.454 | 0.654 | 0.021 | 0.530 |
| PsyCap | -0.040 | 0.017 | 0.021 | 0.068 | 0.020 | 0.052 |
| HP | 0.035 | 0.034 | 0.302 | 0.561 | 0.044 | 0.422 |
| Step 3 |  |  |  |  |  |  |
| C/D×PsyCap | -0.064 | 0.018 | <0.001 | 0.002 | 0.025 | 0.011 |
| C/D×HP | -0.050 | 0.047 | 0.285 | 0.561 | 0.051 | 0.326 |
| PsyCap×HP | 0.022 | 0.032 | 0.503 | 0.654 | 0.049 | 0.662 |
| Step 4 |  |  |  |  |  |  |
| C/D×PsyCap×HP | -0.116 | 0.052 | 0.028 | 0.072 | 0.059 | 0.050 |

Covariates: Sex, Age, SLE, SES, and BMI; SLE: Stressful life events; C/D: Ln C/D ratio; PsyCap: psychological capital; HP: Harsh parenting; **p* <0.05; ** *p* <0.01.

**TABLE 6** The interactions among HCC, psychological capital, and harsh parenting in predicting internalizing problems.

| **Predictors** | ***ΔR^2^*** | ***B*** | ***SE*** | ***β*** | ***95%CI*** | ***p-value*** | ***p_FDR_*** |
| --- | --- | --- | --- | --- | --- | --- | --- |
| Step 1 | 0.3218** |  |  |  |  |  |  |
| Sex |  | 0.101 | 0.029 | 0.182 | [0.043, 0.159] | 0.001 | 0.005 |
| Age |  | -0.007 | 0.022 | -0.017 | [-0.050, 0.036] | 0.737 | 0.817 |
| SLE |  | 0.204 | 0.025 | 0.459 | [0.155, 0.254] | <0.001 | <0.001 |
| SES |  | -0.006 | 0.006 | -0.047 | [-0.018, 0.007] | 0.360 | 0.668 |
| BMI |  | 0.001 | 0.005 | 0.013 | [-0.008, 0.010] | 0.797 | 0.817 |
| Step 2 | 0.037* |  |  |  |  |  |  |
| HCC |  | -0.015 | 0.021 | -0.039 | [-0.056, 0.026] | 0.470 | 0.764 |
| PsyCap |  | -0.042 | 0.017 | -0.142 | [-0.076, -0.008] | 0.016 | 0.052 |
| HP |  | 0.043 | 0.034 | 0.074 | [-0.024, 0.111] | 0.206 | 0.445 |
| Step 3 | 0.014 |  |  |  |  |  |  |
| HCC×PsyCap |  | -0.055 | 0.023 | -0.130 | [-0.100, -0.010] | 0.016 | 0.052 |
| HCC×HP |  | -0.011 | 0.046 | -0.012 | [-0.102, 0.081] | 0.817 | 0.817 |
| PsyCap×HP |  | 0.016 | 0.033 | 0.026 | [-0.048, 0.080] | 0.623 | 0.817 |
| Step 4 | 0.0120* |  |  |  |  |  |  |
| HCC×PsyCap×HP |  | -0.121 | 0.059 | -0.112 | [-0.237, -0.006] | 0.040 | 0.104 |

Covariates: Sex, Age, SLE, SES, and BMI; SLE: Stressful life events; HCC: Ln HCC; PsyCap: psychological capital; HP: Harsh parenting; **p* <0.05; ** *p* <0.01.

The results showed that the interaction between Ln HCC and psychological capital was marginally significant after FDR correction (*p_FDR_* = 0.052). Given this marginal effect, we probed the interaction using simple slope analysis on an exploratory basis, with conditional effects estimated at −1 *SD*, the mean, and +1 *SD* of psychological capital. After FDR correction, the HCC significantly and negatively predicted internalizing problems among adolescents with high psychological capital (*slope* = −0.066, *t* = −2.431, *p*_FDR_ = 0.047), but not at the mean (*slope*= −0.015, *t* = −0.179, *p*_FDR_ = 0.473) and low (*slope* = 0.035, *t* = 1.095, *p*_FDR_ = 0.412) levels. These results are presented in Figure 3.

**FIGURE 2**

Interaction among the Ln HCC, and psychological capital in predicting adolescent internalizing problems. PsyCap refers to psychological capital.

**TABLE 7** The interactions among DHEA, psychological capital, and hash parenting in predicting internalizing problems.

| **Predictors** | ***ΔR^2^*** | ***B*** | ***SE*** | ***β*** | ***95%CI*** | ***p-value*** | ***pFDR*** |
| --- | --- | --- | --- | --- | --- | --- | --- |
| Step 1 | 0.325** |  |  |  |  |  |  |
| Sex |  | 0.094 | 0.029 | 0.170 | [0.036, 0.151] | 0.001 | 0.010 |
| Age |  | -0.011 | 0.022 | -0.027 | [-0.054, 0.032] | 0.607 | 0.816 |
| SLE |  | 0.209 | 0.026 | 0.472 | [0.159, 0.260] | <0.001 | <0.001 |
| SES |  | -0.005 | 0.006 | -0.043 | [-0.018, 0.007] | 0.408 | 0.816 |
| BMI |  | 0.003 | 0.005 | 0.031 | [-0.006, 0.012] | 0.550 | 0.816 |
| Step 2 | 0.027* |  |  |  |  |  |  |
| DHEA |  | -0.016 | 0.033 | -0.026 | [-0.082, 0.049] | 0.628 | 0.816 |
| PsyCap |  | -0.042 | 0.017 | -0.141 | [-0.076, -0.007] | 0.017 | 0.056 |
| HP |  | 0.032 | 0.034 | 0.055 | [-0.036, 0.099] | 0.353 | 0.816 |
| Step 3 | 0.022* |  |  |  |  |  |  |
| DHEA×PsyCap |  | 0.102 | 0.035 | 0.158 | [0.033, 0.170] | 0.004 | 0.017 |
| DHEA×HP |  | 0.011 | 0.078 | 0.008 | [-0.142, 0.165] | 0.885 | 0.959 |
| PsyCap×HP |  | 0.001 | 0.033 | 0.001 | [-0.063, 0.065] | 0.982 | 0.982 |
| Step 4 | 0.000 |  |  |  |  |  |  |
| DHEA×PsyCap×HP |  | -0.053 | 0.093 | -0.032 | [-0.237, 0.131] | 0.569 | 0.816 |

Covariates: Sex, Age, SLE, SES, and BMI; SLE: Stressful life events; HCC: Ln HCC; PsyCap: psychological capital; HP: Harsh parenting; **p* <0.05; ** *p* <0.01.

The results showed that the interaction between Ln DHEA and psychological capital was significant after FDR correction (*p_FDR_* = 0.017). The significant two-way interaction was probed using simple slope analysis, with conditional effects estimated at −1 *SD*, the mean, and +1 *SD* of psychological capital. After FDR correction, the DHEA marginally predicted internalizing problems among adolescents with low psychological capital (*slope* = −0.106, *t* = −2.238, *p*_FDR_ = 0.078), but not at the mean (*slope* = −0.013, *t* = −0.391, *p*_FDR_ = 0.696) and high (*slope* =0.080, *t* = 1.793, *p*_FDR_ = 0.111) levels.
